# Association of depressive symptoms, gait and dynamic balance with risk of falling in older adults discharged from the emergency department after a fall: a prospective observational study

**DOI:** 10.1186/s12877-026-07670-w

**Published:** 2026-05-21

**Authors:** Felix Kastler, Laura Himmelmann, Elisa-Marie Speckmann, Michel Hackbarth, Nina Marie Schmidt, Tania Zieschang, Tim Stuckenschneider

**Affiliations:** Department for Health Services Research, Geriatric Medicine, School VI - School of Medicine and Health Services, Carl von Ossietzky University, Ammerländer Heerstraße 114-118, Lower Saxony 26129 Oldenburg, Germany

**Keywords:** Aged, Prospective falls, Gait analysis, Sensor-based, Margin of stability, Mediation analysis, Principal component analysis, Falls prevention

## Abstract

**Background:**

Depressive symptoms are an established fall-risk factor in older adults, potentially mediated by gait and dynamic balance impairments. However, this relationship remains unexamined in older individuals discharged from the emergency department (ED) following a fall – a group at particularly high risk for recurrent falls. Therefore, this study examined whether depressive symptoms predict 12-month prospective fall risk in this population, and whether gait and dynamic balance impairments act as mediators.

**Methods:**

This prospective sub-study analysed participants from the SeFallED study aged ≥ 60 years who had recently been discharged from the ED following a fall. Data were collected through home visits, gait analyses, and monthly follow-up interviews. Depressive symptoms were assessed using the Depression in Old Age Scale. Gait speed, stride length, swing and double support time were combined into a single gait performance factor using principal component analysis, while dynamic balance was quantified by mediolateral and anteroposterior margin of stability. Ordinal logistic regression analysis investigated the association between depressive symptoms and fall risk, while causal mediation analysis examined gait and dynamic balance as mediators.

**Results:**

In a sample of 143 participants, depressive symptoms (OR 3.74, 95% CI 1.35–10.37, *p* = 0.011) and higher mediolateral margin of stability (OR 1.36, 95% CI 1.07–1.73, *p* = 0.011) independently predicted higher prospective fall risk, whereas gait performance was negatively associated with fall risk (OR 0.47, 95% CI 0.28–0.80, *p* = 0.005). Neither gait nor dynamic balance impairments significantly mediated the association between depressive symptoms and falls.

**Conclusions:**

Depressive symptoms, alongside gait and dynamic balance impairments, represent modifiable indicators of future fall risk following ED presentation after a fall. Integrating mood and mobility assessments into post-ED falls prevention strategies could help mitigate fall risk in this high-risk population.

**Trial Registration:**

Prospectively registered on 4 November 2021 in the Deutsches Register für Klinische Studien, (DRKS00025949; Date of registration in DRKS: 2021–11 – 04).

## Background

In Germany, approximately 24% of adults over the age of 65 fall at least once annually, with the incidence rising to about 33% among those aged 80 years or older [1]. Falls can have severe consequences, including fall-related injuries, subsequent functional impairments, hospitalisation, institutionalisation and death [2]. In 2021, the Global Burden of Disease study attributed nearly 44 million disability-adjusted life-years to falls worldwide [3]. Moreover, the economic burden of falls on global healthcare systems is substantial and continues to grow with demographic aging [4].

Given the significant individual and societal burden of falls, identifying and managing fall risk factors is essential. Depressive symptoms, which affect around 19% of older individuals in Europe and North America, represent one such risk factor [5, 6]. A meta-analysis of 20 studies reported an odds ratio of 1.46 for falls among older individuals with depressive symptoms [7]. Additionally, large longitudinal studies not only demonstrated a bidirectional relationship between depressive symptoms and falls, but also that an increase in depressive symptoms further elevates fall risk [8, 9].

Although the association between depressive symptoms and falls is well established in community-dwelling older adults, little is known about this relationship in older patients discharged from the emergency department (ED) after a fall – a group at particularly high risk for recurrent falls [10]. Moreover, depressive symptoms in older adults are frequently underdiagnosed in primary care and linked to a reduced use of preventive services [11, 12]. Since the ED often serves as the initial point of healthcare contact following a fall [13], targeting this potential high-risk group in the ED could improve access and enable more effective prevention strategies for these patients. However, it remains unclear whether depressive symptoms significantly influence future fall risk in older adults presenting to the ED after a fall.

Gait impairments are frequently observed in older adults with depressive symptoms and may contribute to an increased risk of falling in this population [13, 14]. A systematic review of 20 studies reported that depressive symptoms are associated with slower gait speed, shorter stride length and increased swing and double support time in older adults [16]. These findings were corroborated by another study that assessed spatiotemporal gait parameters in 35 older adults using motion-capture systems [17]. While these studies primarily focused on the association between depressive symptoms and gait alterations, the identified gait impairments are also independently associated with elevated fall risk [18–20]. Additionally, preliminary evidence suggests that gait speed may mediate the relationship between depressive symptoms and falls, with MacAulay et al. (2022) demonstrating that slowed gait speed mediated the relationship between depressive symptoms and fall history in a sample of 147 older adults from an outpatient memory clinic [21].

In addition to gait, depressive symptoms are also linked to impaired balance in older adults [16], with most prior studies focussing on static balance [22–24]. In contrast, data on dynamic balance in older adults with depressive symptoms are limited, despite its central role in maintaining stability during walking [25]. Dynamic balance is commonly quantified using the margin of stability (MoS), which represents an instantaneous measure of mechanical stability of the body during walking [26]. The MoS has been used in previous studies to characterise stability capabilities and coping strategies in individuals with various disorders [25].

Since gait and balance impairments are frequently discussed as potential mechanism linking depressive symptoms to increased fall risk in older adults, ED presentation after a fall would offer an opportunity to assess these impairments and initiate targeted intervention strategies. However, it remains unclear whether gait and dynamic balance impairments mediate future fall risk in older adults with depressive symptoms discharged from the ED after a fall.

This study addressed these gaps in the literature by investigating the association between depressive symptoms and 12-month prospective fall risk in older adults immediately discharged from the ED after a fall. In line with evidence shown earlier, we hypothesised that the presence of depressive symptoms would be associated with an increased risk of future falls in this population. Additionally, as the role of gait and balance is unclear, we explored mechanistic pathways by testing whether gait and dynamic balance mediate the relationship between depressive symptoms and subsequent falls. We hypothesised that both gait and dynamic balance act as mediators explaining part of the effect of depressive symptoms on fall risk.

## Methods

### Participants

This prospective sub-study analysed participants from the SeFallED (“Sentinel Fall Presenting to the Emergency Department”) study, conducted at the Carl von Ossietzky University Oldenburg, Germany. The SeFallED study’s primary objective was to identify long-term functional trajectories of older adults presenting to the ED after a fall without hospital admission. The study protocol has been published previously [27]. The study has been prospectively registered in the German Clinical Trial Register (DRKS-ID: 00025949), approved by the Medical Ethics Committee of the University of Oldenburg (number 2021–120) and conducted in accordance with the Declaration of Helsinki.

Participants were recruited between November 2021 and December 2023 [28]. Inclusion criteria were (1) age ≥ 60 years, (2) presentation to the ED of the Klinikum Oldenburg or Evangelisches Krankenhaus Oldenburg after a fall and discharge within 72 h and (3) informed consent. Exclusion criteria included: (1) life expectancy less than 3 months, (2) unstable medical, neurological or psychiatric condition, (3) bedridden or being unable to walk without support of another person, (4) residence more than 40 km away from the research centre, (5) acute psychosis or social aggression, (6) inability to communicate verbally in German or English.

### Data assessment

We analysed baseline and 12-month follow-up data from the observational part of the SeFallED study, collected at the participants’ homes and in the gait laboratory of the University of Oldenburg. Home visits took place within four weeks after ED presentation and comprised a standardised comprehensive geriatric assessment battery. Ideally one week later, participants attended the gait laboratory for detailed gait analyses. Individuals completely dependent on walking aids or subjectively unable to walk 400 m were ineligible for gait analyses and excluded from this sub-study. During the subsequent 12-month follow-up period, falls were prospectively recorded via monthly telephone interviews, supplemented by fall calendars. Fall incidence was analysed both as a continuous count and categorical variable (no falls, one fall, ≥ 2 falls).

### Measures

#### Depressive symptoms

Depressive symptoms were assessed during the home visit using the Depression in Old Age Scale, which evaluates mood over the preceding two weeks via ten yes/no questions [29]. Total score ranges from 0 to 10 points, with a cut-off ≥ 3 indicating probable depressive symptoms. For analysis, this cut-off score was used to generate a binary variable representing the presence of depressive symptoms (yes/no).

#### Gait parameters

**Fig. 1:**
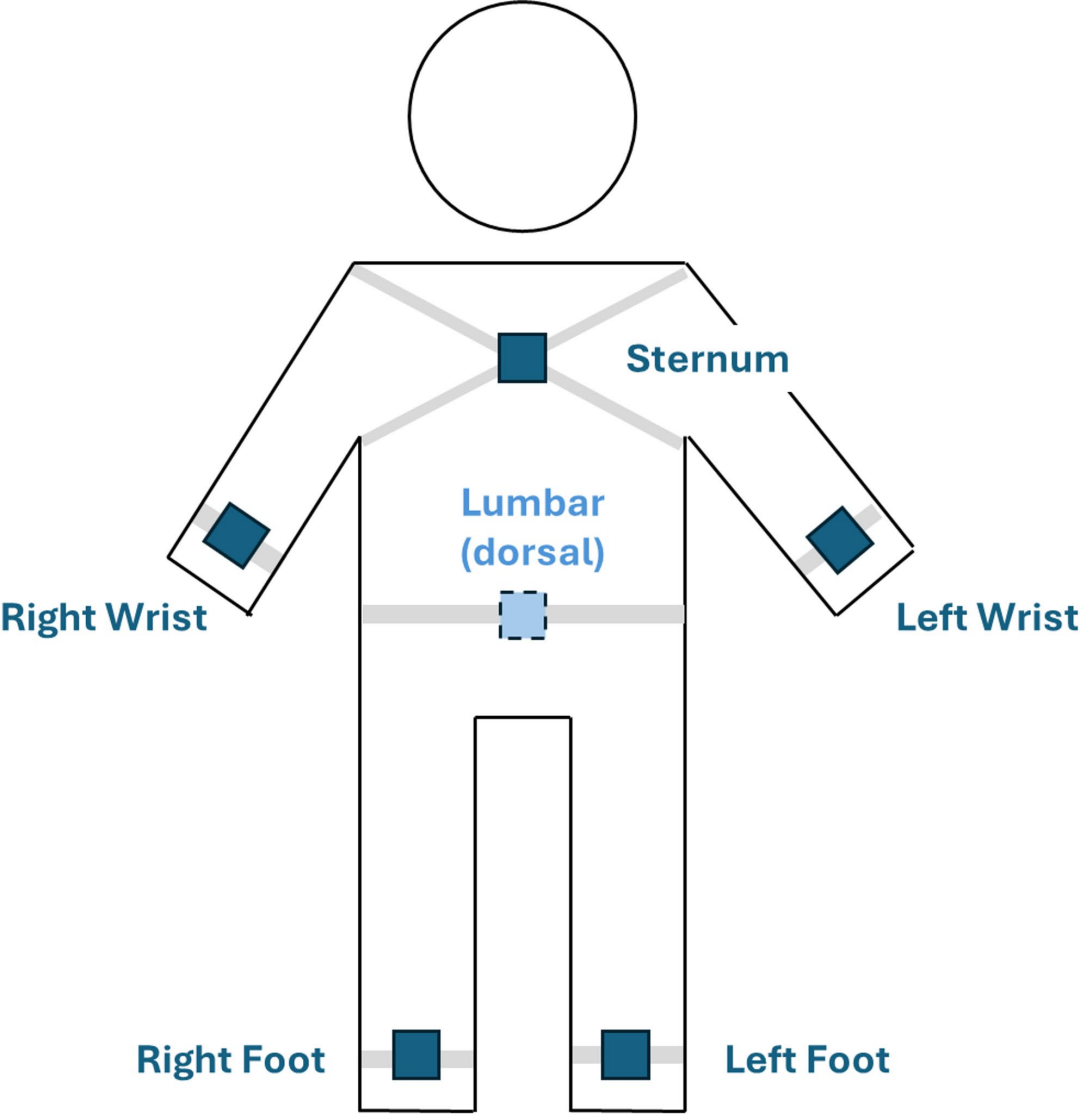
Schematic illustration of the placement of the six inertial measurement units

In the gait laboratory, six wireless, synchronised inertial measurement units (IMU; Opal V1, Mobility Lab™ (ML), APDM, Inc., Portland, OR, USA) were used to capture gait parameters. A schematic illustration of IMU placement is provided in Fig. 1. Preferred overground walking speed was first determined using the mean walking time of two trials over a 3-meter distance. One meter was added to each end of the walkway to minimize the effect of starting, stopping and turning. Participants then walked on a M-Gait treadmill (Motek Medical B.V., Amsterdam, Netherlands) while secured by a safety harness. To minimize potential interference with natural gait patterns, the safety harness did not provide any body-weight support during walking and was adjusted only to passively prevent participants from falling in case of loss of balance. For familiarisation, treadmill speed started at 50% of the preferred overground walking speed and increased by 5% every 30 s until reaching either 100% of the overground walking speed or an individually preferred speed. After six minutes of familiarisation, IMU data from the final minute were used for gait analysis. Gait analyses were terminated upon participants request or if participants reported discomfort, fatigue, or lack of confidence during treadmill walking.

We extracted gait parameters that were linked to depressive symptoms and fall risk in previous studies: gait speed (m/s), stride length (m), swing time (percentage of the gait cycle time; %GCT) and double support time (%GCT) [16–20]. Left and right foot measures were averaged to generate a single mean for each parameter.

#### Margin of stability

Dynamic balance was quantified using the MoS, which was measured with treadmill-embedded force plates and calculated from ground-reaction data using the method of Buurke et al. (2023) [30]. MoS (cm) was defined as the minimal signed distance between the boundaries of the base of support (BoS; represented by the combined centre of pressure) and the extrapolated centre of mass (xCoM; combined position and velocity of the centre of mass) [26]. It was measured at the time of terminal contact in both the anteroposterior (AP-MoS) and mediolateral (ML-MoS) direction while accounting for the relative motion of the treadmill.

#### Covariables

The following covariables were recorded during the home visit: age, sex, antidepressant use (yes/no), number of pre-existing diagnoses (including joint replacements) and number of falls in the 12 months preceding ED presentation. Global cognitive function was assessed using the total score of the Montreal Cognitive Assessment (MoCA; 0–30 points), with higher scores indicating better cognitive status [31]. Concerns about falling were measured with the total score of the Short Falls Efficacy Scale-International (FES-I), comprising seven items rated on a 4-point Likert scale (1 = not at all concerned; 4 = very concerned; total score 7–28 points, with higher scores indicating more concerns about falling) [32]. Performance in activities of daily living (ADL) was evaluated using the total score of the Barthel Index (0-100 points), with higher values indicating greater self-reliance [33]. Instrumental activities of daily living (IADL) were measured via the total score of the Lawton IADL Scale (0–8 points) [34]. Lastly, mobility range was assessed with the German Life Space Assessment (LSA-D), which rates mobility frequency across five life-space levels (indoors, immediate surroundings, local neighbourhood, within town, beyond town) during the preceding four weeks, and whether assistant devices or personal help was needed [35]. The total score was used, with higher values indicating greater mobility and independence.

### Statistical analysis

This exploratory analysis included only complete cases without missing data for depressive symptoms, gait parameters, and the prospective fall incidence. Continuous variables were reported as medians with interquartile ranges (IQR) and categorical variables as absolute and relative frequencies. Participant characteristics are presented for the entire cohort and separately for individuals with and without depressive symptoms. Regarding the prospective fall incidence, exploratory group differences were investigated using the Mann-Whitney-U test.

Due to high collinearity between gait speed, stride length, swing time and double support time, principal component analysis (PCA) was performed to reduce covariance. Factors were extracted based on eigenvalues > 1, scree plots and total variance explained.

To examine the association between depressive symptoms and the prospective fall incidence, ordinal logistic regression was used with the three-category fall count (no falls, one fall, two or more falls) as the dependent variable. The main independent variables were the presence of depressive symptoms, the extracted gait factor and both MoS measures. Two models were fitted: Model 1 adjusted for age and sex; Model 2 additionally adjusted for cognitive status, concerns about falling, ADL and IADL performance, life-space mobility, antidepressant use, the number of pre-existing diagnoses and the number of falls in the preceding 12 months. Covariables were selected a priori using two approaches. First, variables were chosen based on their association with fall risk in previous studies, including age, sex, the number of falls in the preceding 12 months, cognitive function, concerns about falling and antidepressant use [2, 6, 36]. Second, ADL and IADL performance as well as life-space mobility were selected because they showed significant baseline group differences between participants with and without depressive symptoms. Age and sex were included as basic covariables in Model 1 since these represent inherent and directly identifiable factors. All covariables were entered simultaneously into each model to control for potential confounding. Simultaneous entry was chosen to ensure that potential confounders selected a priori were retained in both models rather than being excluded by data-driven selection procedures. Results are presented as odds ratios (OR) – interpreted as the odds of being in a higher fall category – with 95% confidence intervals (95% CI). The proportional odds assumption held for all analysed parameters.

Causal mediation analysis was conducted in R using the mediation package by Tingley et al. (2014) to investigate whether gait and dynamic balance impairments mediated the relationship between depressive symptoms and the prospective fall incidence [37]. The presence of depressive symptoms served as the independent variable, whereas the total number of falls during follow-up was used as the continuous outcome variable. Gait factors and MoS measures were included as mediators only if they significantly predicted prospective fall risk in the ordinal logistic regression model. Analyses were adjusted for age and sex. Linear regression modelled paths between depressive symptoms and the mediators, whereas negative-binomial regression was used for paths involving the prospective fall count. Indirect, direct, and total effects were estimated using nonparametric bootstrapping with 5,000 samples to compute 95% CIs. Effects were considered significant when the 95% CI did not include zero.

All analyses were performed in SPSS version 29.0 (IBM Corp., Armonk, NY, USA) and R version 4.5.0 (R Foundation), with significance level set at *p* < 0.05.

## Results

### Participants’ characteristics

**Fig. 2:**
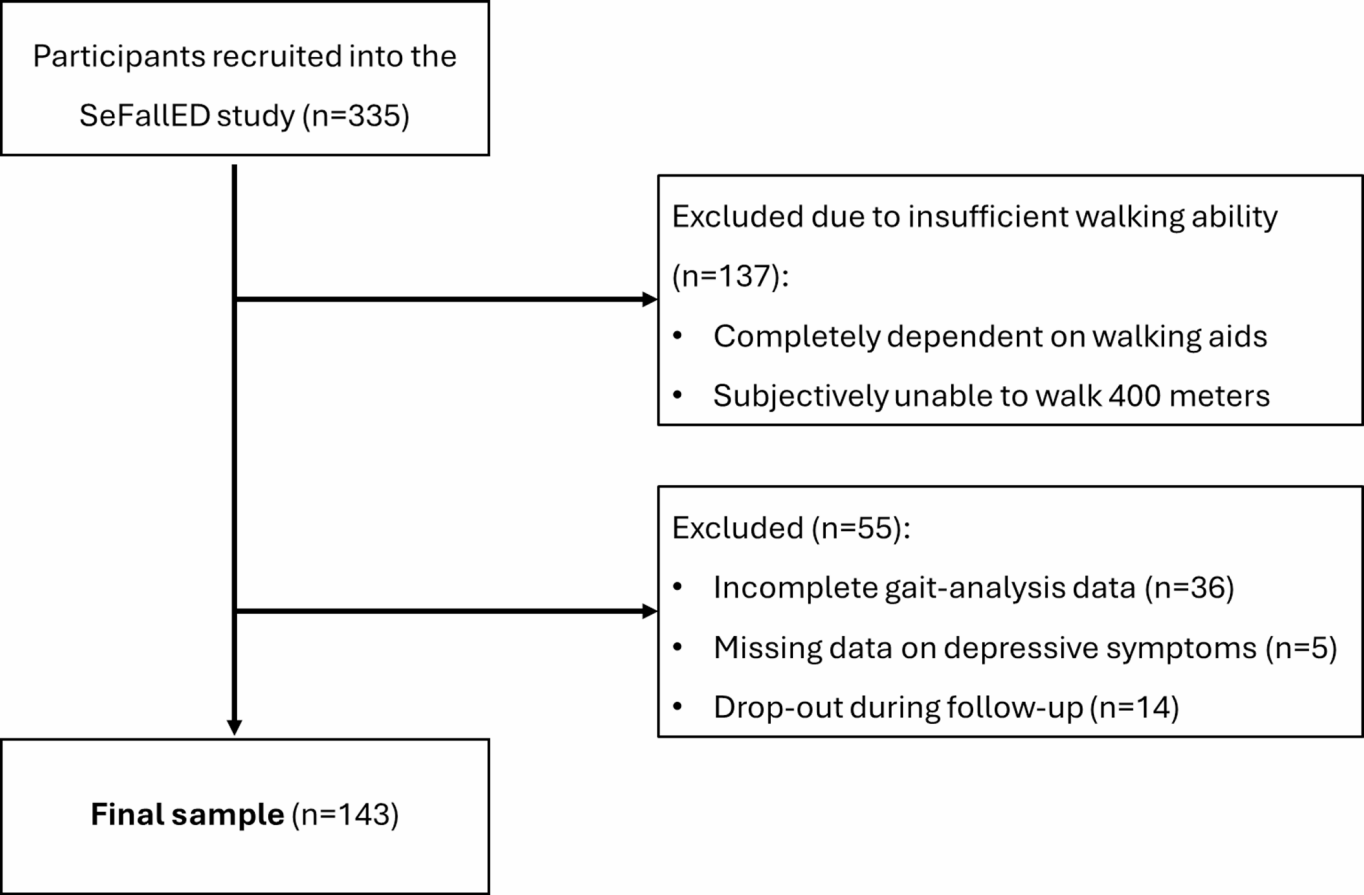
Flowchart representing inclusion and exclusion of participants

Figure 2 illustrates the inclusion and exclusion process for participants. Of the 335 individuals recruited into the SeFallED study, 137 were excluded from this sub-study due to insufficient walking ability. A further 36 were excluded for incomplete gait-analysis data, five lacked information on depressive symptoms, and 14 dropped out during follow-up, resulting in a final sample of 143 participants (median age 71.0 (IQR 66.0–78.0) years, 60.8% females).

The mean interval between the home visit and the subsequent gait analysis was 35.4 days. No falls occurred during gait analysis, and no individuals prematurely terminated the assessment.

Participants’ characteristics are summarised in Table 1. Over 12 months of follow-up, 26.6% of all participants reported one fall, while another 23.8% reported two or more falls. Individuals with depressive symptoms (*n* = 28, 19.6%) experienced a significantly higher number of falls during follow-up compared to those without (median number of falls 1.0 (IQR 0.25–2.75) vs. 0.0 (IQR 0.0–1.0), *p* = 0.001). Participants’ gait parameters and MoS data are summarised in Table 2.

**Table 1:** Participants’ characteristics presented for the total cohort and stratified by depressive symptom status

Characteristics	All participants (*n* = 143)	With depressive Symptoms (*n* = 28)	Without depressive Symptoms (*n* = 115)
Female Sex, n (%)	87 (60.8)	20 (71.4)	67 (58.3)
Age (years)	71.0 (66.0–78.0)	72.0 (67.3–78.0)	71.0 (65.0–78.0)
Barthel Index (score)	100.0 (100.0-100.0)	100.0 (96.2–100.0)	100.0 (100.0-100.0)
Lawton IADL Scale (score)	8.0 (8.0–8.0)	8.0 (8.0–8.0)	8.0 (8.0–8.0)
MoCA (score)	25.0 (23.0–27.0)	25.0 (23.0-27.8)	25.0 (23.0–27.0)
FES-I (score)	8.0 (7.0–10.0)	10.0 (7.3–11.8)	7.0 (7.0–9.0)
LSA-D (score)	85.0 (68.0-100.0)	72.0 (58.1–89.0)	90.0 (72.0-100.0)
Number of pre- existing diagnoses (n)	2.0 (1.0–4.0)	3.0 (1.0–5.0)	2.0 (1.0–3.0)
Number of falls in the preceding 12 months (n)	1.0 (1.0–1.0)	1.0 (1.0–1.0)	1.0 (1.0–1.0)
Individuals using Antidepressants, n (%)	9 (6.3)	6 (21.4)	3 (2.6)
Prospective fall Incidence (n)	1.0 (0.0–1.0)	1.0 (0.25–2.75) ^◊^	0.0 (0.0–1.0) ^◊^
Prospective fall incidence (categorised), n (%)			
0	71 (49.7)	7 (25.0)	64 (55.7)
1	38 (26.6)	8 (28.6)	30 (26.1)
≥ 2	34 (23.8)	13 (46.4)	21 (18.3)

Values are shown as median (interquartile range)

^**◊**^ *p* = 0.001 (exploratory baseline group comparison using Mann-Whitney-U test)

*FES-I* Short Falls Efficacy Scale-International Version, *IADL* instrumental activities of daily living, *LSA-D* Life Space Assessment German Version, *MoCA* Montreal Cognitive Assessment

**Table 2:** Gait parameters and Margin of Stability measures presented for the total cohort and stratified by depressive symptoms status

Gait Parameter	All participants (*n* = 143)	With depressive Symptoms (*n* = 28)	Without depressive Symptoms (*n* = 115)
Gait speed (m/s)	0.88 (0.71–1.07)	0.75 (0.54–1.05)	0.92 (0.73–1.07)
Stride length (m)	0.98 (0.79–1.10)	0.83 (0.66–1.10)	1.00 (0.84–1.10)
Swing time (%GCT)	38.4 (36.4–40.1)	36.9 (35.7–38.5)	38.7 (36.8–40.1)
Double support time (%GCT)	23.4 (19.9–27.2)	26.2 (23.1–28.5)	22.8 (19.7–26.4)
Mediolateral Margin of Stability (cm)	4.4 (3.0-5.7)	3.7 (2.2–5.8)	4.5 (3.1–5.7)
Anteroposterior Margin of Stability (cm)	6.5 (-1.1-11.1)	6.4 (1.2–11.7)	6.6 (-1.6-11.0)

Values are shown as median (interquartile range)

*%GCT* percentage of the gait cycle time

### Principal component analysis

Results of the PCA are shown in Table 3. The Kaiser-Meyer-Olkin measure of sampling adequacy was 0.716, representing an adequate factor analysis. Bartlett’s test of Sphericity was significant (*p* < 0.001), indicating sufficiently large correlation between items. Based on eigenvalues, scree plots and theoretical considerations, a one-factor solution was chosen, with no rotation applied. All four gait parameters loaded highly on this factor, which accounted for 88.7% of the total variance and was named gait performance. As shown in Table 3, higher gait performance factor scores represent faster gait speed, longer stride length, increased swing time and reduced double support time.

**Table 3:** Item loading for the one-factor solution obtained from principal component analysis (no rotation applied)

	Gait performance
Gait speed (m/s)	0.952
Stride length (m)	0.913
Swing time (%GCT)	0.952
Double support time (%GCT)	-0.950
Variance explained (%)	88.7

*%GCT* percentage of the gait cycle time

### Association between depressive symptoms and falls

Table 4 displays the results of the ordinal logistic regression analysis. Model fit indices indicated a modest improvement in model fit after inclusion of the additional covariables in Model 2 compared to Model 1 (Model 1: χ^2^ = 42.75, df = 6, *p* < 0.001, Nagelkerke Pseudo-R^2^ = 0.312, AIC = 253.19 vs. Model 2: χ^2^ = 55.70, df = 14, *p* < 0.001, Nagelkerke Pseudo-R^2^ = 0.394, AIC = 251.92). Depressive symptoms and gait performance were independently associated with higher prospective fall risk during follow-up in Model 1 (depressive symptoms: OR 3.69, 95% CI 1.51–9.04, *p* = 0.004; gait performance: OR 0.44, 95% CI 0.28–0.69, *p* < 0.001) and Model 2 (depressive symptoms: OR 3.74, 95% CI 1.35–10.37, *p* = 0.011; gait performance: OR 0.47, 95% CI 0.28–0.80, *p* = 0.005 ). Additionally, higher ML-MoS was significantly associated with prospective fall risk in both models (Model 1: OR 1.34, 95% CI 1.07–1.67, *p* = 0.010; Model 2: OR 1.36, 95% CI 1.07–1.73, *p* = 0.011).

**Table 4:** Ordinal logistic regression analysis investigating the association between depressive symptoms and fall risk

Predictor	Model 1		Model 2	
	OR (95% CI)	*p*-value	OR (95% CI)	*p*-value
Depressive symptoms (yes/no)	3.69 (1.51–9.04)	0.004	3.74 (1.35–10.37)	0.011
Gait performance	0.44 (0.28–0.69)	< 0.001	0.47 (0.28–0.80)	0.005
ML-MoS (cm)	1.34 (1.07–1.67)	0.010	1.36 (1.07–1.73)	0.011
AP-MoS (cm)	1.02 (0.96–1.08)	0.543	1.04 (0.98–1.10)	0.247
Age (years)	1.04 (0.98–1.10)	0.201	1.05 (0.99–1.12)	0.102
Sex (female/male)	1.85 (0.82–4.14)	0.137	1.87 (0.79–4.45)	0.289
Number of pre-existing diagnoses	-	-	0.88 (0.69–1.12)	0.405
Number of falls in preceding 12 months	-	-	0.91 (0.44–1.89)	0.808
Antidepressant use (yes/no)	-	-	1.17 (0.22–6.18)	0.852
Cognitive status (MoCA, total score)	-	-	0.86 (0.73–1.01)	0.060
Concerns about falling (FES-I, total score)	-	-	1.11 (0.97–1.27)	0.142
Life-space mobility (LSA-D, total score)	-	-	1.01 (0.99–1.02)	0.594
ADL performance (Barthel Index, total score)	-	-	0.88 (0.77–1.01)	0.076
IADL performance (Lawton IADL Scale, total score)	-	-	1.71 (0.77–3.80)	0.191

*95% CI* 95% confidence interval, *ADL* activities of daily living, *AP-MoS *anteroposterior Margin of Stability, *FES-I* Short Falls Efficacy Scale-International Version, *IADL* instrumental activities of daily living, *LSA-D* Life Space Assessment German Version, *ML-MoS* mediolateral Margin of Stability, *MoCA* Montreal Cognitive Assessment, *OR* odds ratio for the odds of being in a higher fall category compared to the reference category

### Mediation analysis

**Fig. 3:**
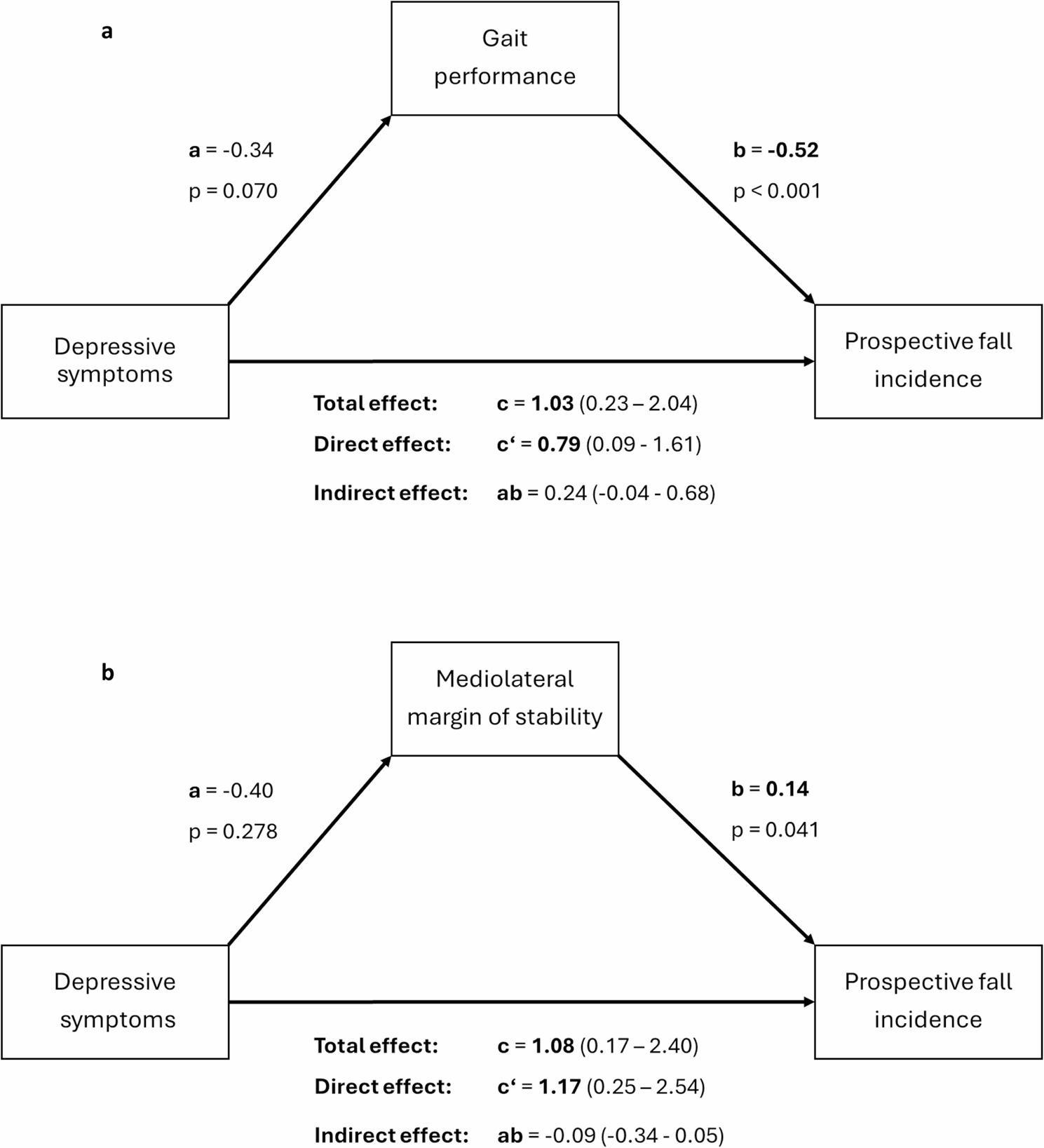
Results of the causal mediation analysis. **a** Mediating effect of gait performance on the relationship between depressive symptoms and prospective fall incidence. **b** Mediating effect of mediolateral margin of stability on the relationship between depressive symptoms and prospective fall incidence. Mediation analysis adjusted for age and sex. Total, direct and indirect effects are presented with 95% confidence intervals

Figure 3a illustrates the results of the causal mediation analysis for gait performance. Without accounting for gait performance, a significant total effect of depressive symptoms on the prospective fall incidence (path c) was observed. After entering gait performance as a mediator into the model, depressive symptoms showed no significant association with gait performance (path a), whereas higher gait performance factor scores were negatively associated with the prospective number of falls (path b). A significant direct effect of depressive symptoms on fall risk remained after mediation (path c’). No significant mediating effect of gait performance on the relationship between depressive symptoms and the prospective fall incidence was observed (indirect effect: ab = 0.24, 95% CI -0.04-0.68). Since AP-MoS was not significantly associated with prospective fall risk, only ML-MoS was entered into a separate mediation model, yielding similar results (see Fig. 3b).

## Discussion

This prospective observational study explored the relationship between depressive symptoms, gait, dynamic balance, and prospective fall risk among older adults recently discharged from the ED following a fall. In line with our first hypothesis, depressive symptoms were associated with higher prospective fall risk during the 12-month follow-up period in the fully adjusted ordinal logistic regression model. Furthermore, greater ML-MoS also independently predicted future fall risk, while gait performance was negatively associated with the risk of falling. However, contrary to our second hypothesis, neither gait performance nor dynamic balance mediated the association between depressive symptoms and prospective falls in our population.

PCA yielded a single factor, termed gait performance, with high loadings from all four gait parameters. In contrast, previous studies using PCA to analyse gait in older adults – often including larger sample sizes and a broader range of gait parameters – reported multiple factors [18, 38]. In our analysis, the inclusion of a small number of strongly intercorrelating gait variables likely contributed to the emergence of only a single factor. Notably, the loadings of gait speed, stride length, swing and double support time on our factor correspond to those reported previously [18, 38]. Therefore, our findings support the use of a single factor to reduce collinearity and summarise gait performance in our sample. However, future research should include a broader range of gait parameters – for example measures of gait variability and asymmetry – to further characterize gait patterns and their influence on fall risk in older adults with depressive symptoms.

The prevalence of depressive symptoms in our sample was 19.6%, which is consistent with a meta-analysis of 25 studies of older adults in Western countries [5]. Depressive symptoms were significantly associated with increased prospective fall risk, independent of age, sex, gait performance, dynamic balance, and additional fall risk factors. This finding aligns with previous longitudinal studies in community-dwelling older adults. Choi et al. (2019) reported that baseline depressive symptoms predicted multiple falls over one year in 6,299 older adults, even after adjustment for fall history, concerns about falling, demographic and health-related factors [8]. Similarly, Song et al. (2023) found a significant association between depressive symptoms and elevated fall risk over two years in a cohort of 14,356 older adults, adjusted for fall history, sociodemographic and health-related factors [9]. Together with previous research, our findings highlight the importance of assessing depressive symptoms as a marker of future fall risk in older adults presenting to the ED after a fall. Since depressive symptoms are a potentially modifiable fall risk factor, they should be considered in falls prevention strategies following ED presentation. Addressing depressive symptoms may, therefore, serve a dual purpose by not only improving mental health but also reducing fall risk in this high-risk population.

Gait performance – representing gait speed, stride length, swing and double support time – was negatively associated with future fall risk in our study, corroborating previous research in older adults [18–20]. Regarding dynamic balance, higher ML-MoS, but not AP-MoS, was independently associated with prospective fall risk in both models. These findings align with the results of Lencioni et al. (2021), who reported that higher ML-MoS, but not AP-MoS, correlated with fall history in individuals with neurological disorders [38]. Theoretically, higher ML-MoS indicates greater dynamic balance, since the xCoM is further medial from the lateral boundaries of the BoS [26]. However, prior studies have interpreted shifting the xCoM away from the BoS as a potential compensatory strategy for maintaining balance in fall-prone individuals [25, 38, 39]. Kazanski et al. (2024) further hypothesised that increased mean ML-MoS in older adults may reflect greater gait variability, which is counteracted by taking wider steps, thus, increasing ML-MoS [40]. Comparable mechanisms likely underlie the association between higher ML-MoS and fall risk observed in our sample. To better clarify the interplay between gait, dynamic balance and fall risk, future studies should include additional gait measures, like step width and gait variability, and combine force-plate data with motion-capture systems, which represents the gold standard for gait analysis [41].

In the causal mediation analysis, depressive symptoms did not significantly predict gait performance, whereas gait performance was negatively associated with prospective fall risk, and a significant direct effect of depressive symptoms on fall risk remained. Consequently, no significant mediating effect of gait performance on the relationship between depressive symptoms and falls was observed. These findings contrast with results from Mac-Aulay et al. (2022), who showed that slower gait speed mediated the relationship between depressive symptoms and fall history in 147 older adults presenting to an outpatient memory clinic for cognitive complaints [21]. Notably, our study examined prospective fall risk in older individuals presenting to the ED after a fall, whereas MacAulay et al. assessed falls retrospectively in a more specific sub-group, limiting direct comparability. Moreover, multiple interacting mechanisms likely underlie the relationship between depressive symptoms and falls in older adults [13, 14]. Major pathways include concerns about falling, deficits in attention and executive function, impaired sleep, weight loss and adverse effects of antidepressant medications. Several of these mechanisms may increase fall risk partly by affecting gait and balance. For example, concerns about falling are associated with impairments in gait speed, stride length and double support time [42]. Similarly, deficits in attention and executive function in older adults with depressive symptoms are linked to slower gait speed and greater unsteadiness [15], while antidepressant use contributes to fall risk in part via impaired balance and drug-induced movement disorders [43]. Although some of these variables – such as concerns about falling and cognitive status – were measured in the present study, they showed no significant association with fall risk in the fully adjusted ordinal logistic regression model. However, this does not necessarily exclude their potential role as mediators or moderators in more complex causal pathways. The presence of such additional factors may explain, why no significant effect of depressive symptoms on gait performance and no significant mediating effect was observed. Due to limited sample size, more complex statistical approaches – such as structural equation models testing for multiple parallel or serial mediators and moderators – were not feasible. Future studies with larger sample size should, therefore, consider such multivariate models to more comprehensively evaluate pathways between depressive symptoms, gait and falls in older adults.

Although no mediating effects were found, our results highlight the independent predictive value of depressive symptoms alongside gait and balance impairments for prospective fall risk in our population. Interventions addressing both domains are, therefore, warranted. Physical exercise may be a promising approach, as it can positively influence both depressive symptoms as well as gait and balance in older adults [44, 45]. Future research should focus on the combined effects of such interventions on mood and fall risk and consider integrating similar approaches into post-ED falls prevention.

Even though our study provides novel insights into the relationship between depressive symptoms, gait, dynamic balance and falls in a high-risk population, certain limitations must be acknowledged. First, only participants with sufficient walking ability were included, which reduced sample size and statistical power. Additionally, our inclusion criteria likely produced an overrepresentation of highly functional participants — reflected in overall ADL and IADL scores — creating a ceiling effect, which increased susceptibility to bias. Future studies should consider less demanding mobility assessments to include a broader spectrum of functional capacities, permitting larger, more representative samples and a clearer understanding of the relationship between depressive symptoms and future falls in this high-risk group. With the rapid advancement of wearable technology, real-life monitoring may soon become feasible, allowing the inclusion of individuals with lower levels of physical functions [46].

Second, differences in the timing of mood, gait and dynamic balance assessments may have reduced the reliability of our analysis. Depressive symptoms were assessed during a home visit within four weeks of ED discharge, whereas gait and dynamic balance were evaluated in the gait laboratory, ideally one week later. Because recovery trajectories following a fall vary substantially between individuals, these timing differences may have influenced both depressive symptom status and mobility performance at the time of measurement. The four-week interval between ED presentation and the home visit was deliberately chosen to allow sufficient recovery for participation while still capturing early post-fall trajectories. However, logistical and ethical constraints required flexible scheduling of both home visits and subsequent gait assessments. Although the gait laboratory was intended to occur approximately one week after the home visit, the mean interval between assessments was 35.4 days. This temporal variability may have attenuated the exposure-mediator relationship; therefore, the results of our causal mediation analysis should be interpreted as exploratory. Future studies should incorporate repeated assessments of mood and mobility to better account for temporal variability in post-fall recovery.

Third, depressive symptoms were dichotomised for analysis, which reduced statistical power compared to analysing continuous depressive symptom scores [49]. Dichotomisation may also explain imprecise estimations observed for depressive symptoms, as reflected by the OR and wide 95% CI.

Fourth, gait analyses were performed under controlled laboratory conditions, with participants walking on a treadmill at their preferred speed while secured by a harness. This setting does not reflect everyday walking with varying surfaces, inclines and frequent starting and stopping, thus limiting generalisability. Additionally, the short 3-meter walkway used to calculate preferred overground walking speed resulted in a limited number of strides, potentially causing underestimation of this measure. Nonetheless, this approach allowed safe and standardised gait assessments in our high-risk population. Future research employing wearable sensors could elucidate the influence of depressive symptoms on real-world daily walking in older adults.

Fifth, the number of predictors included in the fully adjusted ordinal logistic regression model is borderline relative to our sample size, which may increase the risk of overfitting and reduce the stability of coefficient estimates. However, this approach allowed us to control for multiple potential confounders relevant for fall risk in older adults. Future studies with larger sample sizes aimed at identifying the strongest predictors of future fall risk following a fall-related ED presentation should consider data-driven approaches, like stepwise regression models, to optimize model fit. Also, participants’ pre-existing comorbidities were analysed as a cumulative count, which limited the ability to assess the impact of specific conditions on gait and fall risk. Given the clinical heterogeneity of our study population, future research should consider more detailed comorbidity assessments, like the Charlson comorbidity index, to better characterise potential confound effects.

Lastly, while the gait performance factor derived from PCA allowed us to explore the multidimensional nature of gait and to reduce multicollinearity among highly correlated gait parameters, it lacks clinical interpretability. Consequently, effect estimates derived from this factor cannot be expressed in clinically meaningful units (such as m/s) or attributed to specific gait components. Because a single composite factor was used to represent gait, individual gait parameters such as gait speed – which have been widely studied and for which clinically relevant cut-offs exist [47, 48] – were not analysed separately. Future studies with larger sample sizes should aim to unravel gait performance by analysing individual gait parameters, thereby identifying clinically relevant cut-off metrics and better characterising the specific mechanisms linking depressive symptoms, gait and fall risk in older adults.

Among this study’s strengths was its longitudinal design, enabling the identification of the predictive value of depressive symptoms, gait, and dynamic balance for fall risk. Data were obtained through multimodal assessments using validated instruments, which enabled adjustment for multiple potential confounders in our analyses. Additionally, systematic prospective fall documentation via telephone interviews and fall calendars improved data quality by reducing recall bias. Finally, focusing on older adults discharged from the ED after a fall addressed a high-risk population underrepresented in current research due to the complexity of enrolling older patients in the ED environment [28]. Prospective fall incidence was high, with half of the participants experiencing at least one fall during follow-up, underscoring the need for targeted intervention strategies for this population.

## Conclusion

This exploratory study identified depressive symptoms alongside gait and balance impairments as independent, modifiable indicators of future fall risk among older adults discharged from the ED after a fall. Ideally, ED-based assessments should incorporate evaluations of both depressive symptoms as well as gait and balance to capture their combined influence on fall risk over time. However, the feasibility of such assessments in the acute and often resource-limited ED environment remains unclear. Given the findings and limitations of our study, larger-scale investigations that account for multiple mechanisms potentially affecting fall risk are warranted to clarify the role of depressive symptoms in older adults presenting to the ED following a fall. Targeted interventions addressing both mood and mobility may ultimately help reduce falls and improve outcomes in this high-risk population.

## Data Availability

The datasets used and analysed during the current study are available from the corresponding author upon reasonable request. As public availability would compromise patient privacy, storing the data in a public repository is not possible. However, upon request and after approval by the local ethics committee, it may be made available. To receive data, please contact the corresponding author.

## Abbreviations

ED: Emergency department
MoS: Margin of Stability
IMU: Inertial measurement unit
%GCT: Percentage of the gait cycle time
BoS: Base of support
xCoM: Extrapolated centre of mass
AP-MoS: Anteroposterior margin of stability
ML-MoS: Mediolateral margin of stability
MoCA: Montreal Cognitive Assessment
FES-I: Short Falls Efficacy Scale-International
ADL: Activities of daily living
IADL: Instrumental activities of daily living
LSA-D: German Life Space Assessment
IQR: Interquartile range
PCA: Principal component analysis
OR: Odds ratio
95% CI: 95% confidence interval
BMFTR: German Federal Ministry of Research, Technology and Space
